# Recombinant Peptide Mimetic NanoLuc Tracer for Sensitive Immunodetection of Mycophenolic Acid

**DOI:** 10.1021/acs.analchem.1c02109

**Published:** 2021-07-14

**Authors:** Álvaro Luque-Uría, Riikka Peltomaa, Tarja K. Nevanen, Henri O. Arola, Kristiina Iljin, Elena Benito-Peña, María C. Moreno-Bondi

**Affiliations:** † Department of Analytical Chemistry, Faculty of Chemistry, Complutense University, Ciudad Universitaria s/n, 28040 Madrid, Spain; ‡ VTT Technical Research Centre of Finland Ltd, Tietotie 2, FI-02150 Espoo, Finland

## Abstract

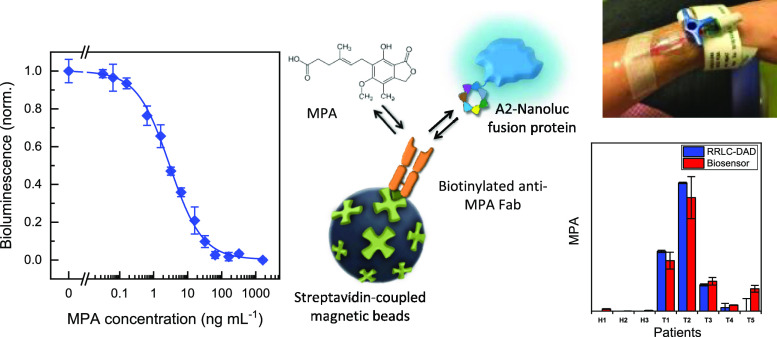

Mycophenolic acid (MPA) is an immunosuppressant drug commonly used to prevent organ rejection in transplanted patients. MPA monitoring is of great interest due to its small therapeutic window. In this work, a phage-displayed peptide library was used to select cyclic peptides that bind to the MPA-specific recombinant antibody fragment (Fab) and mimic the behavior of MPA. After biopanning, several phage-displayed peptides were isolated and tested to confirm their epitope-mimicking nature in phage-based competitive immunoassays. After identifying the best MPA mimetic (ACEGLYAHWC with a disulfide constrained loop), several immunoassay approaches were tested, and a recombinant fusion protein containing the peptide sequence with a bioluminescent enzyme, NanoLuc, was developed. The recombinant fusion enabled its direct use as the tracer in competitive immunoassays without the need for secondary antibodies or further labeling. A bioluminescent sensor, using streptavidin-coupled magnetic beads for the immobilization of the biotinylated Fab antibody, enabled the detection of MPA with a detection limit of 0.26 ng mL^–1^ and an IC_50_ of 2.9 ± 0.5 ng mL^–1^. The biosensor showed good selectivity toward MPA and was applied to the analysis of the immunosuppressive drug in clinical samples, of both healthy and MPA-treated patients, followed by validation by liquid chromatography coupled to diode array detection.

## Introduction

Mycophenolic acid (MPA) is a mycotoxin produced by *Penicillium* fungi, and it is widely used as an immunosuppressant drug to prevent organ rejection in transplanted patients.^1^ Recently, it has also been tested as a chemotherapeutic agent as it inhibits the proliferation of cancer cells.^2^ Due to the small therapeutic window that MPA has, it is very important to monitor correctly its levels inside the human body.^3^ MPA is mainly found in the serum, but only 1% of the total MPA exists in the free form, which is the one responsible for its pharmacological activity.^3,4^ Therefore, the availability of analytical methods for detecting MPA at low concentrations in serum is of great interest.

Over the past decades, the determination of MPA has been carried out using liquid chromatography (LC) coupled with ultraviolet or mass spectrometry detection.^5−7^ However, these methods often require skilled personnel and they are time-consuming and of high cost. Moreover, tedious sample treatment is mandatory in most cases. Fast screening methods such as immunoassays are highly relevant nowadays, and the use of antibodies has burst over the last years as simple analytical tools. Immunoassays offer outstanding versatility since they can be easily automated or integrated into a routine laboratory or a point-of-care testing device. Also, different immunoassays have been already implemented for the detection of MPA.^8−10^ Those assays, however, fail to detect free MPA in blood samples and offer a poor selectivity as several potential interferences may alter the results. We have previously developed a homogeneous fluorescence polarization assay to detect free MPA in blood samples with good sensitivity, low cross-reactivity, and good recovery rates in real samples.^11^

The analysis of low molecular weight molecules can sometimes be challenging. They might present high toxicity, carcinogenicity, high price, or are difficult to functionalize without altering their interaction with the antibody. A feasible solution to this is the use of peptide mimetics, also known as mimotopes, since they can be easily functionalized or fused to other proteins in a cost-effective way. Peptide mimetics have the exceptional ability to bind to the same antibody paratope as the antigen, and they can be applied to the development of competitive immunoassays or biosensors where they can replace the analyte conjugate used as the competitor.

Phage display is a commonly applied technique for recombinant antibody development as well as to identify peptide mimetics.^12^ Phage-based enzyme-linked immunosorbent assays (ELISA) using peptide mimetics have been widely described in the literature. These assays do not require much preparation, and they have good sensitivity as well as selectivity.^13−17^ However, the presence of phage may have a significant effect on the binding kinetics, and previous reports have shown that the assay sensitivity can potentially improve when the peptide is used alone rather than in the phage-displayed form.^18,19^ Moreover, the assays would be faster, cheaper, and simpler if the peptide is fused to a fluorescent or luminescent protein, since the peptide fusion would be responsible for the analytical signal, and there would be no need of using any secondary antibody for that purpose. The coupling can typically be a genetic fusion or a chemical functionalization; however, the former one is preferred due to the fact that chemical modifications can lead to a series of secondary reactions that may alter the final product. Genetic modifications are more homogeneous and present a well-defined stoichiometry between the peptide and the protein.^20^

In this work, we describe the first peptide mimetic for MPA and a bioluminescent-based immunoassay for the detection of MPA with a NanoLuc–peptide fusion in blood samples. First, the peptide mimetic was selected from a combinatorial peptide library by phage display. The high selectivity of the peptide mimetic for the recombinant MPA antibody fragment was demonstrated by a competitive phage-based ELISA. Moreover, surface plasmon resonance (SPR) was used to confirm the binding properties of the cyclic peptide (named A2) and MPA to the anti-MPA Fab antibody. Thereafter, a bioluminescent protein, NanoLuc, was coupled to the MPA mimicking peptide A2. NanoLuc is reported to be 100 times brighter than firefly or *Renilla* luciferases, and with a size as small as 19 kDa, it is catching the eyes of many researchers for many different applications.^21^ The NanoLuc–peptide fusion was genetically crafted and implemented in a magnetic bead-based immunoassay that showed higher sensitivity than the phage-based ELISA. Finally, the bioluminescent assay was applied to analyze the free active forms of MPA in blood samples from transplanted patients. The results were validated by a reference method using rapid resolution LC with diode array detection (RRLC-DAD).

## Experimental Section

### Materials

The Ph.D.-C7C Phage Display Peptide Library Kit was purchased from New England Biolabs (Ipswich, MA, USA). Nunc MaxiSorp 96-well plates, Amplex UltraRed reagent, Phusion Hot Start II DNA Polymerase, High-Fidelity DNA Polymerase, SuperBlock blocking buffer [in phosphate-buffered saline (PBS)], LB Broth, Lennox, Human serum type AB, EZ-Link Sulfo-NHS-LC-Biotin, No-Weigh Format, 1-Step ultra TMB-ELISA, and NeutrAvidin Biotin Binding Protein were from Thermo Fisher Scientific (Waltham, MA, USA). Streptavidin microtiter plates were from Kaivogen (Turku, Finland). Polymerase chain reaction (PCR) Nucleotide Mix and 2,2′-azino-di-(3-ethylbenzthiazoline sulfonic acid) (ABTS) were purchased from Roche Diagnostics (Basel, Switzerland). Black Packard HTRF 96-well plates were from Nunc (Roskilde, Denmark), and the biotinylated peptide A(CEGLYAHWC)GGGSK(Bio)-NH_2_ was synthesized at Peptide Synthetics (Fareham, UK). The horseradish peroxidase (HRP)-conjugated anti-M13 antibody, HisTrap FF crude columns, Sephadex G-25 M columns, and Illustra NAP-5 columns were purchased from Cytiva (Chicago, IL, USA). Cobalt(II) chloride hexahydrate (for analysis) and hydrogen peroxide 30% were obtained from Merck (Darmstadt, Germany). PBS, pH 7.4, Tween 20, dimethyl sulfoxide (≥99.5%), 5-bromo-4-chloro-3-indolyl β-d-galactopyranoside (X-Gal), and isopropyl-β-d-thiogalactopyranoside (IPTG) were purchased from Sigma-Aldrich (Saint Louis, MO, USA). LB Agar and Agar Granulated were from NZYtech (Lisbon, Portugal), and imidazole and MPA were purchased from Alfa Aesar (Maverhill, MA, USA). BcMag IDA-modified magnetic beads (1 μm) were from Bioclone Ltd. (London, UK). PCR primers were purchased from Integrated DNA Technologies, Inc (San Diego, CA, USA). NanoGlo Reagent for Immunoassay was from Promega Corporation (Madison, WI, USA), and High Capacity Magne Streptavidin Beads and ATG-42 plasmid DNA, containing the NanoLuc gene, were kindly donated by Promega Corporation (Madison, WI, USA). The recombinant anti-MPA Fab was obtained from a phage display library and produced as described previously.^22^

### Biopanning Rounds

A commercial phage-displayed peptide library was used to select cyclic peptides that bind to the anti-MPA. The selection rounds were carried out with an automatic magnetic bead processor (KingFisher Thermo Fisher Scientific). See the Supporting Information for antibody coupling to magnetic beads. Briefly, the phage-displayed peptide library (∼2.0 × 10^11^ phages) was incubated for 2 h with the anti-MPA conjugated beads (50 μg) in a total volume of 505 μL of PBST [PBS, pH 7.4 with 0.05% (v/v) Tween-20]. The beads were subsequently washed twice with PBST for 30 s, and then the bound phages were eluted with 100 μL of 0.1 M triethylamine (pH 11.2) for 30 min. The resulting solution containing the eluted phages was immediately neutralized with 70 μL of 1 mol L^–1^ Tris-HCl (pH 6.8). Amplification of the eluted phages was carried out by adding 70 μL of the eluate to a 40 mL early-log phase ER2738 culture in LB and incubating at +37 °C for 4.5 h. The cells were harvested by centrifugation (10 min, 12,000*g*, +4 °C), and the supernatant was collected. The amplified phages were precipitated overnight at +4 °C after adding to the supernatant 1/6 volume of 20% poly(ethylene glycol) (PEG)/2.5 mol L^–1^ NaCl. Then, the precipitated phages were collected by centrifugation (15 min, 12,000*g*, +4 °C) and resuspended in 3 mL of PBS. The precipitation was repeated with 20% PEG/2.5 mol L^–1^ NaCl on ice for 1 h, followed by centrifugation (10 min, 12,000*g*, +4 °C). Finally, the pellet containing the phages was resuspended in 500 μL of PBS. The amplified phage solution was utilized for the consequent selection round.

After the first round, an additional 30 s washing step was introduced to harden the conditions of selection. After three panning rounds, several individual clones were isolated from each round and tested in phage-based ELISAs to select the one showing the highest sensitivity for the anti-MPA. Monoclonal phages were selected from fresh titering plates of each round. Briefly, 80 μL of ER2738 culture containing the monoclonal phages were incubated for 2.5 h at +37 °C and were subsequently streaked out and grown overnight on IPTG/X-Gal plates at 37 °C. Afterward, individual clones were inoculated on 500 μL of LB and grown for 6 h at +37 °C. Finally, the cells were harvested (5 min, 10,000*g*, +4 °C), and the supernatant was transferred to a fresh tube. The concentration of the amplified individual clones, determined by tittering, ranged from 10^11^ to 10^12^ pfu mL^–1^.

### Phage-Based ELISA

The phage-displayed peptides were screened in an ELISA to test their binding to immobilized anti-MPA. The assay was carried out at room temperature (RT). The biotinylated anti-MPA (Supporting Information) [5 μg mL^–1^ in the assay buffer (SuperBlock supplemented with 0.05% Tween-20); 100 μL per well] was immobilized on streptavidin-coated wells (30 min), followed by three-time washes with PBST. The wells were then blocked with 280 μL of assay buffer for 30 min and washed again three times with PBS. Then, the amplified phage stock (between 10^10^ and 10^11^ pfu mL^–1^; 100 μL per well) was added to the wells in assay buffer and incubated for 1 h with slow shaking. After washing the wells as described above, the HRP-conjugated anti-M13 monoclonal antibody (1:5000 dilution in assay buffer; 100 μL per well) was added to the wells and incubated for 1 h. Finally, the plate was washed three times as described above and 100 μL of ABTS was added to the wells. After 5 min, absorbance at 405 nm was measured in a Varioskan plate reader (Thermo Scientific).

The phage clone that showed binding to the anti-MPA Ab was tested in a similar assay in the presence of 100 ng mL^–1^ of free MPA. Furthermore, a bead-based assay was developed with the phage that showed significant competition in the plate-based assay. Briefly, black microtiter plates were blocked with 280 μL of assay buffer for 1 h at RT and subsequently washed three times with PBS. Then, the biotinylated anti-MPA (1.2 μg mL^–1^) and neutravidin-coated magnetic beads (125 μg mL^–1^) functionalized as described before,^18^ were added to the wells in the assay buffer (total volume 260 μL per well), and incubated for 30 min at RT. After washing the beads using a plate washer with a magnetic support, the phage clone (10^11^ pfu mL^–1^) and increasing concentrations of free MPA were added to the wells (in assay buffer, 60 μL per well) and incubated for 30 min at RT. The beads were washed again to remove the excess, followed by incubation with HRP-conjugated anti-M13 antibody (1:5000 dilution in assay buffer; 80 μL per well) for 30 min at RT. Finally, after washing, 80 μL of Amplex UltraRed solution was added to each well, and the fluorescence was monitored with a CLARIOstar microplate reader (BMG Labtech) (λ_ex_ = 530 nm and λ_em_ = 590 nm).

### Construction of the NanoLuc Fusion Protein

The phage clone that showed the best response in the competition assay with free MPA was sequenced to identify the peptide sequence. To express the MPA peptide mimetic A2 in fusion with the NanoLuc protein, the latter one was PCR-amplified from the commercial vector ATG 42^23^ using the Phusion Hot Start II DNA Polymerase. The forward primer, RP043, (5′-GAA AAC CTG TAT TTT CAG GGC GTC TTC ACA CTC GAA GAT TTC G-3′) hybridized to the 5′-end of the NanoLuc, and the reverse primer, RP044, (5′-ATA CAG ACC CTC ACA ACT GCC ACC TCC AGA GCC GCC ACC CGC CAG AAT GCG TTC GC-3′) hybridized to the 3′-end. The hybridizing part of the sequence is underlined. The fusion of NanoLuc with the cyclic peptide was carried out in the pMAL vector. In order to amplify this vector, the forward primer, RP039, (5′-GT **TGT GAG GGT CTG TAT GCG CAT TGG TGC GGA GGC**TAG GGA TCC GAA TTC CCT-3′) included a 5′-overhang (in bold) for the DNA sequence encoding the peptide mimetic for MPA, whereas the reverse primer, RP040, (5′-G AAA ATA CAG GTT TTC ATG ATG ATG ATG ATG ATG CAT AAT CTA TGG TCC TTG TTG G-3′) contained a His-tag. For the assembly, the vector and the insert were incubated at +50 °C for 15 min with the NEBbuilder Master Mix. Then, NEB 5-alpha competent *Escherichia coli* cells were transformed with 2 μL of the assembled product according to the manufacturer’s instructions.^24^ Successful cloning was proven by DNA sequencing analysis.

### Expression and Purification of the Fusion Protein

The A2-NanoLuc plasmid (Figure S1A, Supporting Information) was first transformed into *E. coli* SHuffle Express cells according to the manufacturer’s instructions. A single colony was selected on LB agar plates with 100 μg mL^–1^ ampicillin and grown on 15 mL of LB with 100 μg mL^–1^ ampicillin overnight. The next day, an aliquot of the overnight preculture was added to a 200 mL culture of LB with 100 μg mL^–1^ ampicillin and grown until an OD_600_ (optical density at 600 nm) of 0.6 was reached. To induce the protein expression, IPTG was added at a final concentration of 0.4 mmol L^–1^, and the expression was continued at +37 °C for 4 h. The culture was then transferred to an ice bath for 10 min to stop the cell growth, and the cells were collected by centrifugation at 5000*g* for 10 min at +4 °C and resuspended in NZY Bacterial Cell Lysis Buffer (approximately 5 mL of buffer per gram of cell paste) supplemented with a protease inhibitor cocktail, NZY Bacterial Cell Lysis Buffer supplemented with Lysozyme and DNase I according to the manufacturer’s instructions. The cell lysis was carried out by sonication (VibraCell Ultrasonic Processor 130 W 20 kHz, Ampl 70%) for 10 s 5 times with 30 s breaks, and the insoluble cell debris was discarded by centrifugation at 15,000*g* for 15 min at +4 °C. Finally, the cell lysate was purified with HisTrap purification columns according to the manufacturer’s instructions, and the buffer was exchanged to PBS with Sephadex G-25 M columns. The purified proteins were aliquoted and stored at −20 °C. The size and purity of the A2-NanoLuc fusion protein was confirmed by sodium dodecyl sulfate–polyacrylamide gel electrophoresis (Figure S1B Supporting Information). The kinetic constants of the binding of the cyclic peptide (A2) and MPA were determined by Biacore T200 (GE Healthcare) (Supporting Information).

### Bioluminescent Immunoassay for MPA Detection

**Figure 1:**
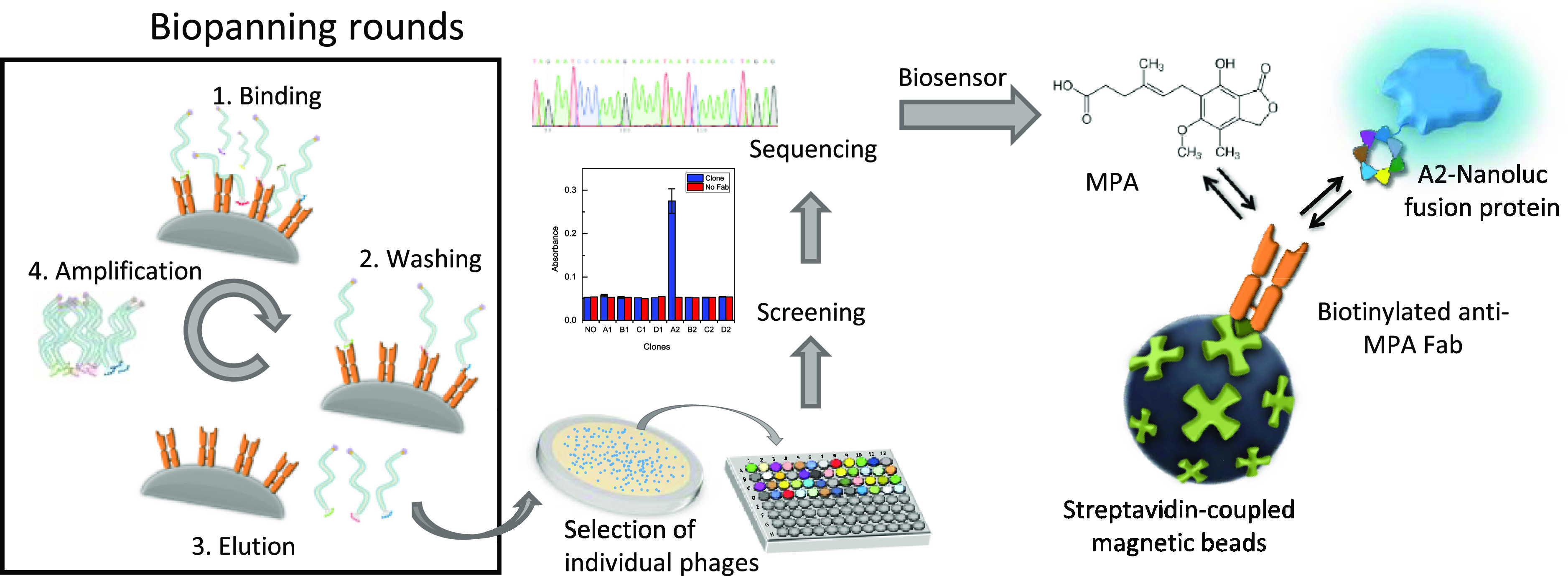
Schematic representation of the biopanning rounds, followed by the whole process until the biosensor development for the detection of MPA based on the A2-NanoLuc fusion protein. MPA concentration was determined by a competition between the free MPA and A2-NanoLuc for the binding sites of the biotinylated anti-MPA Fab antibody, previously bound to streptavidin-coupled magnetic beads. Finally, the bioluminescence of NanoLuc was measured after the addition of the NanoGlo substrate.

To detect MPA with the A2-NanoLuc fusion protein, a bead-based assay was carried out on a black microtiter well plate by immobilizing the biotinylated anti-MPA onto streptavidin-coated magnetic beads (Figure 1). Briefly, the wells were first blocked with assay buffer (SuperBlock with 0.05% Tween-20) for 1 h. Then, 60 μL of 5 μg mL^–1^ biotinylated anti-MPA in assay buffer and 20 μL of streptavidin beads (1:50 dilution from the stock) were added to the wells and incubated for 30 min at RT. After washing three times with PBST, 60 μL of a solution containing different concentrations of MPA and 77 μg mL^–1^ of the A2-NanoLuc in assay buffer was added to the wells and incubated 30 min at RT. Once the beads were washed, 60 μL of NanoGLO substrate in PBS were added and bioluminescence was measured after a 2 min incubation at 470 nm with a bandwidth of 80 nm using a CLARIOstar microplate reader.

### Sample Analysis

Volunteers donated whole blood samples with permission from the Ethics Committee from Hospital Clínico Universitario de Valladolid, Spain (no. PI 21-2245). The blood samples were kept at 20 °C during transport and storage. The samples were treated following the procedure described previously (see the Supporting Information for details).^11^

## Results and Discussion

### Selection and Characterization of MPA Peptide Mimetics

**Figure 2:**
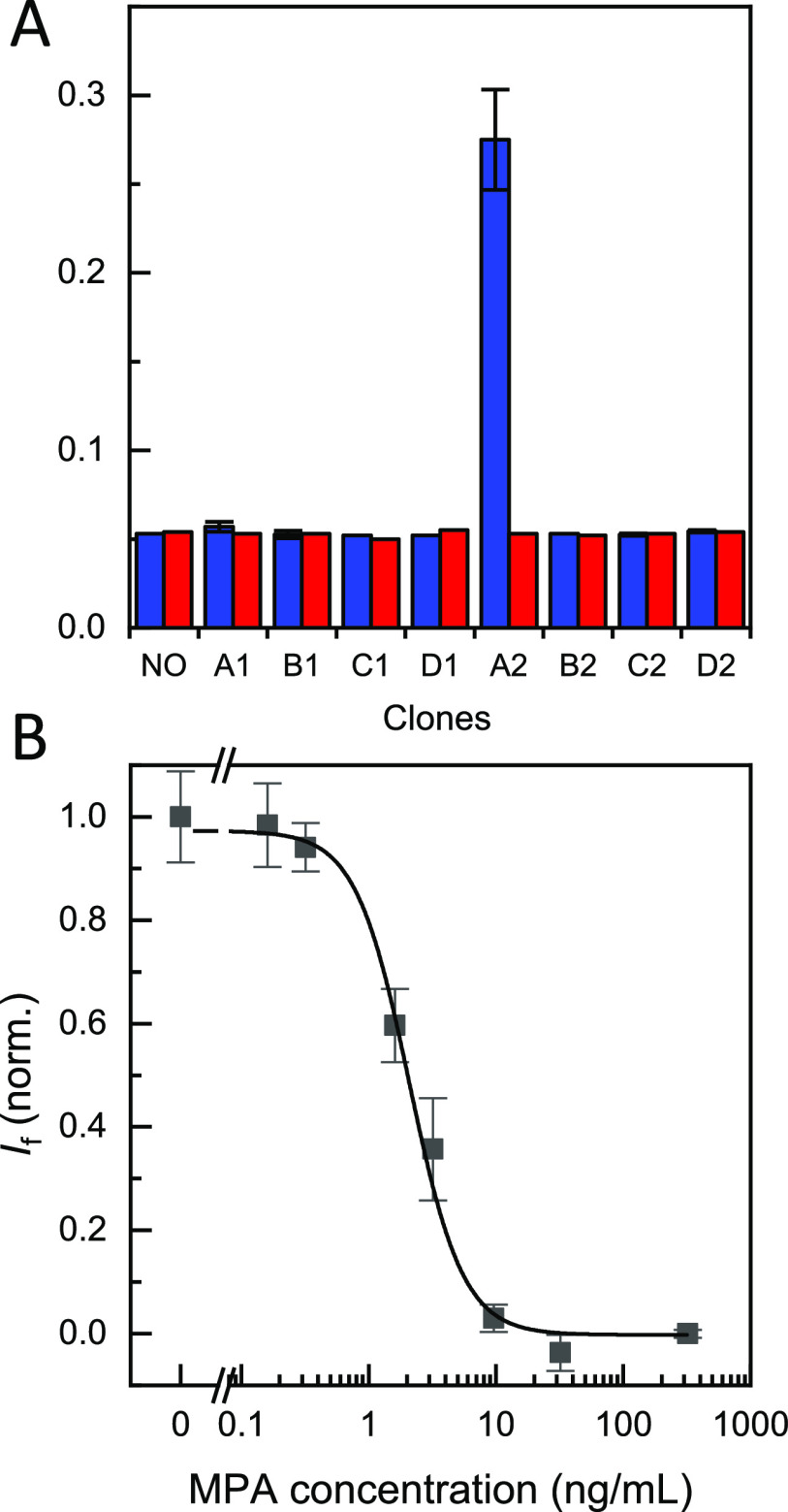
(A) Phage-based ELISA with eight different monoclonal phages. Clone A2 showed high specificity toward anti-MPA (blue) and very low nonspecific binding in the absence of anti-MPA (red), similar to the background wells (NO clone). (B) Competitive phage-based ELISA with clone A2. Free MPA was added simultaneously with the phage clone A2 to the wells containing the anti-MPA immobilized onto magnetic beads in assay buffer. The results are shown as the average fluorescence intensity ± the standard error of the mean (*n* = 3). The response was fitted to a logistic fit using OriginPro 2019.

To develop a competitive immunoassay for MPA detection, a peptide mimetic for MPA was selected from a cyclic 7-mer phage display peptide library (Ph.D.-C7C) in three consecutive panning rounds. Once the three panning rounds were carried out, a total of eight clones were isolated and tested using ELISA. One of the clones showed a very high signal-to-background ratio, as well as very low nonspecific binding when the assay was performed in the absence of anti-MPA (Figure 2A); therefore, this clone (named A2) was selected for further analysis. Next, a competitive ELISA for A2 was carried out under the same assay conditions as before. However, in this case, 100 ng mL^–1^ of free MPA were added at the same time as the phage clone to test the competition between phage-displayed A2 and free MPA for the binding sites of the anti-MPA. A significant decrease in the signal was observed in the presence of MPA, demonstrating the success of the selection rounds and excellent performance of clone A2 as a peptide mimetic (data not shown).

A fluorescent bead-based assay was developed to further optimize the assay conditions and confirm the viability of the selected phage clone. Neutravidin-functionalized magnetic beads were incubated with the biotinylated anti-MPA, and the competition was then tested between free MPA in concentrations ranging from 0 to 1600 ng mL^–1^ and clone A2. The results were similar to those obtained on the plate-based ELISA, confirming the successful selection of the peptide mimetic (Figure 2B).

By DNA sequencing of clone A2, the peptide sequence of **ACEGLYAHWC**, with a disulfide bond between the two cysteines, was identified. A synthetic biotinylated peptide with this sequence was consequently tested in a competitive neutravidin bead-based assay, showing competition at the nanomolar level. Contrary to the phage-based assay, this time, the biotinylated peptide was bound to neutravidin beads, and the nonbiotinylated anti-MPA was added thereafter. This antibody was then recognized with an anti-IgG-HRP antibody, measuring the same fluorescent signal as before. Due to the absence of the whole phage in this assay, the results prove that the peptide sequence obtained can be considered an outstanding mimetic for MPA since a similar response was obtained in comparison to the phage-based assay (Figure S2, Supporting Information). As can be seen, the phage-based assay showed a slightly lower limit of detection (LOD), calculated as the 10% inhibition,^25^ (0.69 ng mL^–1^) compared to the peptide-based assay (0.94 ng mL^–1^). However, the dynamic range, taken as the 20–80% inhibition,^26^ is wider in the case of peptide-based assay (2.4–60 ng mL^–1^) than in phage-based assay (1.0–4.1 ng mL^–1^). The assay time is the same in both cases, and the detection is done by adding the same fluorescent dye.

### Binding Properties of Cyclic Peptide

To compare the binding properties of the biotinylated cyclic peptide and MPA toward the anti-MPA antibody, label-free SPR technology was applied. In the binding experiments, previously identified, produced, and purified Fab antibodies recognizing either MPA or ochratoxin A were immobilized onto sensor chip surfaces.^22^ The same experimental conditions were used to study the binding properties of cyclic peptide (A2) and MPA. The results are presented in Figures S3 and S4 and summarized in Table S1 (Supporting Information). As expected, both cyclic peptide (A2) and MPA showed binding to the anti-MPA Fab antibody surface, and the binding responses increased in a concentration-dependent manner.

In agreement with our previous results from the SPR assay using affinity in solution approach, the affinity constant for MPA and anti-MPA Fab antibody interaction was ∼40 nmol L^–1^.^22^ The affinity of the interaction between cyclic peptide (A2) and anti-MPA Fab antibody is 2 orders of magnitude lower compared to the affinity of MPA–anti-MPA Fab antibody interaction. This is due to the slower association and faster dissociation of cyclic peptide (A2)–anti-MPA Fab antibody complex compared to the corresponding values for the MPA–anti-MPA Fab antibody complex.

### Bioluminescent Bead-Based Immunoassay for MPA Detection

**Figure 3:**
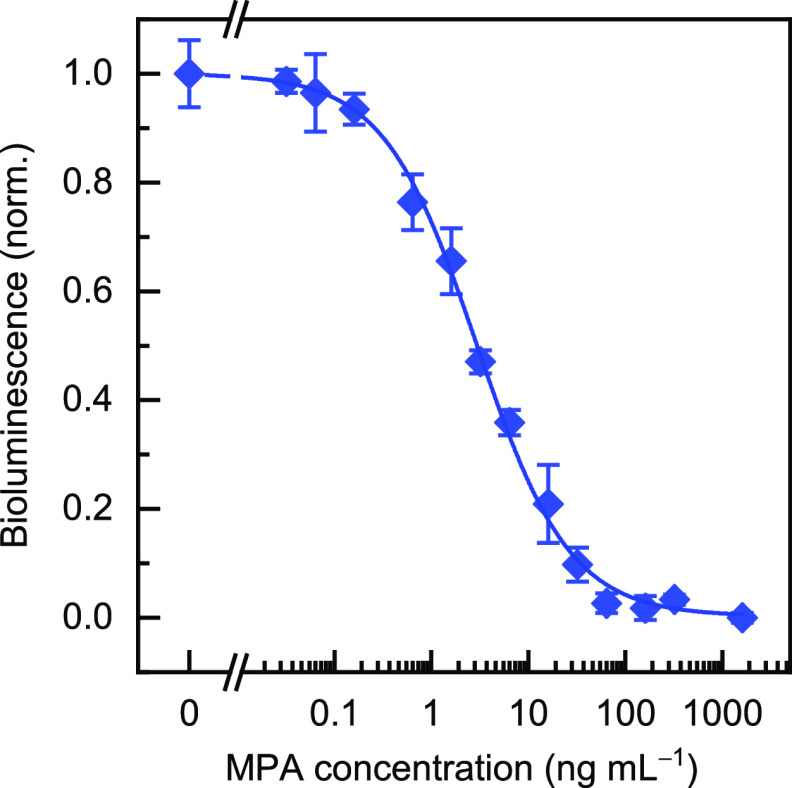
Bead-based bioluminescent MPA calibration in assay buffer using the A2-NanoLuc fusion protein. Different MPA concentrations were incubated with A2-NanoLuc and magnetic beads coupled to the biotinylated anti-MPA. The bioluminescence signals (λ_em_ = 470 ± 80 nm) were measured after adding the NanoGLO substrate, and the values were normalized to the maximum and minimum signals. The results are presented as the mean values ± the standard error (*n* = 3) adjusted to a logistic fit using OriginPro 2019.

To improve the assay sensitivity and to provide a faster and cheaper assay, the peptide mimetic was fused to a bioluminescent enzyme, both in the N-terminus and C-terminus (A2-Nanoluc and NanoLuc-A2, respectively), and a simple immunoassay for MPA detection was established using the A2-NanoLuc fusion protein. The fusion protein was produced cost-effectively by bacteria, in which the bioluminescent protein can be already incorporated. After purification, both NanoLuc-A2 and A2-NanoLuc fusion proteins showed bright luminescence in the presence of the substrate, proving that the assay did not require a secondary antibody or any other chemical modification to obtain the analytical signal. Both fusion proteins also proved to recognize the anti-MPA and compete with free MPA at the nanomolar level for the binding sites of the antibody (Figure S5, Supporting Information); however, the A2-Nanoluc product showed a wider dynamic range and lower dispersity at low concentrations, and it was selected for further characterization (Figure 3). This confirmation was carried out with a bead-based assay, in which streptavidin-coated beads were incubated first with the biotinylated anti-MPA, and then, A2-NanoLuc and free MPA were added simultaneously to the solution. This bead-based immunoassay improved both the dynamic range and the sensitivity compared to similar bead-based assays carried out with the phage-displayed A2 and with the synthetic peptide A2-bio (Figure S2, Supporting Information). The LOD was 0.26 ng mL^–1^ and the IC_50_ value was 2.9 ± 0.5 ng mL^–1^. The dynamic range ranged between 0.64 and 14 ng mL^–1^. The interday relative standard deviation was 12% on average (*n* = 3), whereas the value for assays on three different, nonconsecutive days was 9%. The A2-NanoLuc fusion protein proved to be stable for more than 6 months upon storage at −20 °C in PBS. For comparison purposes, this bioluminescent assay provided a better sensitivity, a shorter analysis time, and simplicity, since there is no need to add a secondary antibody, than those described previously using HRP as the label and fluorometric detection. In addition, the sensitivity of this assay is better than for other immunoassays described in the literature, as well as for several commercially available kits for the analysis of MPA (Table S2, Supporting Information).

### Cross-Reactivity

**Figure 4:**
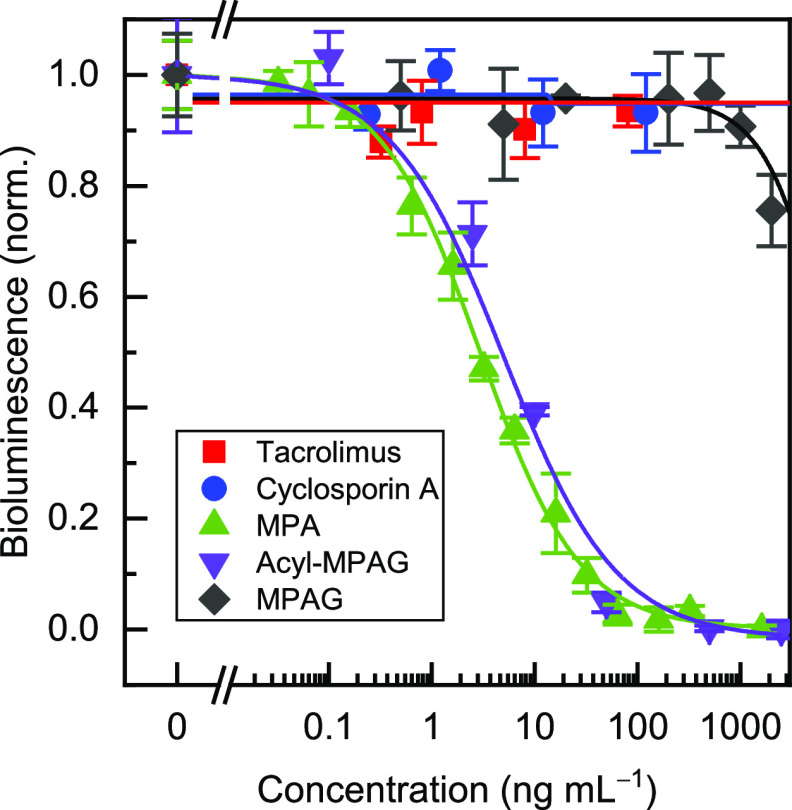
Cross-reactivity of the bead-based bioluminescent immunoassay. MPAG and acyl-MPAG are metabolites that can be found in blood together with MPA. Tacrolimus and cyclosporine are two immunosuppressant drugs that can be administered in combination with MPA to transplanted patients to prevent organ rejection. The bioluminescence values were normalized to the maximum and minimum signals, and the results are presented as the mean values ± the standard error of the mean (*n* = 3) adjusted to a logistic fit using OriginPro 2019.

To prove the selectivity of the method, the assay was performed in the presence of different MPA metabolites found in blood, such as mycophenolic acid glucuronide (MPAG) and acyl-mycophenolic acid glucuronide (acyl-MPAG), as well as other immunosuppressant drugs commonly co-administered to transplanted patients, tacrolimus and cyclosporin (Figure S6 Supporting Information). As can be observed in Figure 4, acyl-MPAG showed a very similar behavior to MPA in the assay (58% cross-reactivity, calculated as the IC_50_ for MPA divided by the IC_50_ of acyl-MPAG). This metabolite is an active form of MPA, contrary to MPAG;^4^ therefore, the assay can be designed to detect the active forms of MPA in blood. Nevertheless, acyl-MPAG is found at lower concentrations than MPA,^27^ and it was not detected by high-performance LC in any of the analyzed samples. Concerning MPAG, the cross-reactivity was negligible at 0.03%, and for the two other immunosuppressant drugs, it was lower than 0.03%.

### Matrix Effect

**Figure 5:**
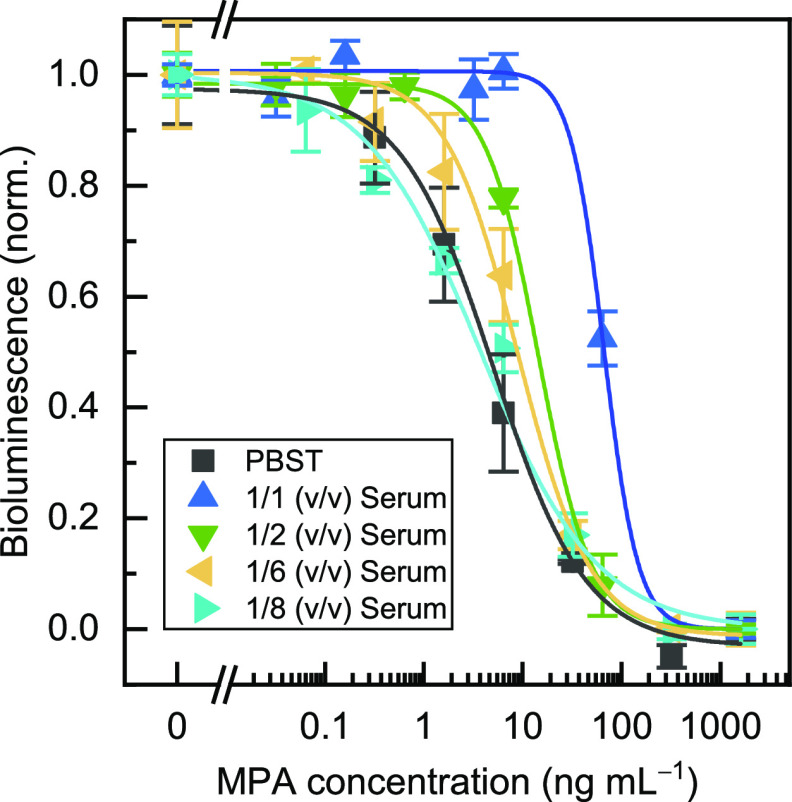
Comparison of the calibration curves for the bead-based bioluminescent immunoassay in PBST and with different dilutions of ultrafiltered serum. No significant differences were found with the 1:8 (v/v) dilution. The bioluminescence values were normalized to the maximum and minimum signals, and the results are presented as the mean values ± the standard error of the mean (*n* = 3). The graph was adjusted to a logistic fit using OriginPro 2019.

The matrix effect was tested in the presence of different dilutions of the ultrafiltered serum samples [1/2, 1/6, and 1/8, (v/v)], treated following a previously described procedure,^11^ in PBST. Figure 5 shows that no significant differences (*p* > 0.05) were observed between the dose response curves obtained in PBST or in an ultrafiltered serum diluted 1/8 (v/v) with the buffer. Therefore, such dilution was used for further experiments.

### Sample Analysis

**Figure 6:**
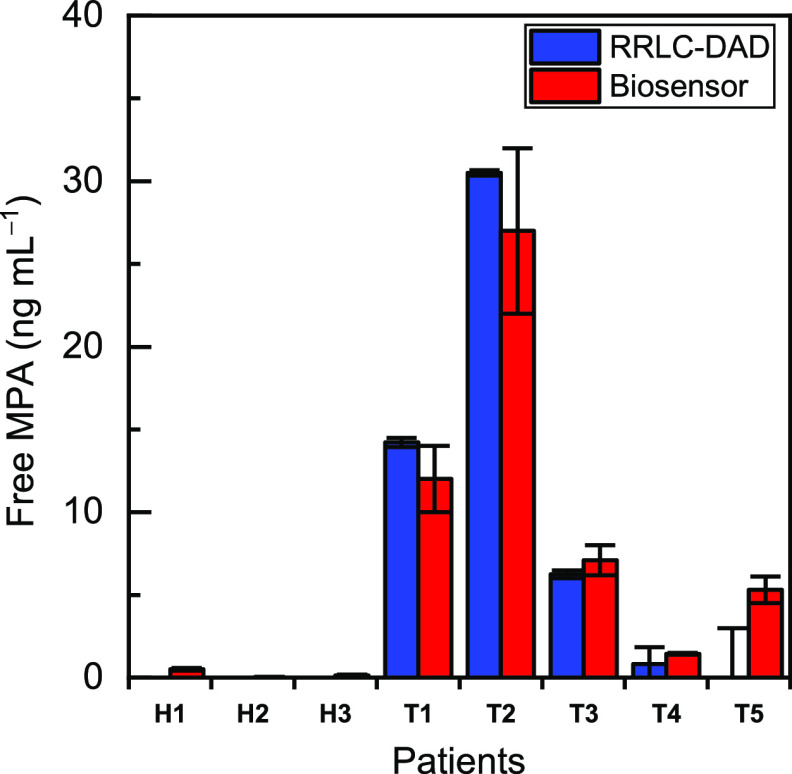
Results of the comparison of the analysis of blood samples from transplanted patients by the biosensor and RRLC-DAD. H refers to healthy patients, not treated with MPA, and T to MPA-treated patients. The results are presented as the mean values ± the standard error of the mean (*n* = 3).

The optimized assay was applied to the analysis of blood samples from transplanted patients (T1–T5) and healthy control patients (H1–H3), and the results were validated by RRLC-DAD (Supporting Information) (Figure 6). Figure S7 (Supporting Information) shows a chromatogram of a standard mixture of the metabolites. As expected, no MPA was detected in the control samples. A statistical comparison of the results obtained by both methods using a paired *t*-test demonstrated that there are no significant differences between them at a 95% confidence level. The RRLC-DAD results confirmed that the active metabolite, acyl-MPAG, was not present in any of the samples, and therefore, the biosensor response was only due to the free MPA. Furthermore, the MPAG levels found in the analyzed samples were below the limit of quantification of the biosensor; hence, the nonactive metabolite of MPA did not cross-react in the analysis (Table S3, Supporting Information). The results show that patients T1 and T2 had the highest MPA concentration levels, and the results in all cases correlate favorably with the administered doses (Table S4, Supporting Information).

## Conclusions

In this work, we proved that phage display is a useful technique for the selection of MPA peptide mimetics for the development of immunoassays and biosensors. A bioluminescent bead-based assay using a luciferase enzyme as a reporter provided higher sensitivities, shorter analysis times, and cost-effective assays than other formats using HRP as the label and fluorometric detection. The assay allows the analysis of the active forms of MPA in plasma, that is, free MPA and acyl-MPAG. No relevant cross-reactivity was observed with other nonactive forms of MPA in plasma as well as with other drugs jointly administered to transplanted patients. The results were compared favorably with a reference RRLC-DAD-based method.

## Supporting Information Available

Protocols for antibody coupling to magnetic beads and antibody biotinylation; details about the synthetic peptide-based ELISA; description about the SPR measurements; details about the RRLC-DAD method; blood sample treatment; construction of the NanoLuc-peptide mimetic fusion; calibrate comparison of both NanoLuc-peptide mimetic fusions; chemical structures of MPA, its main metabolites, and two other co-administered immunosuppressant drugs; comparison of different analytical methods reported for MPA detection; MPAG levels found in transplanted patients; and administered doses of MPA to the analyzed transplanted patients (PDF)
