# Supplementary material for: Recombinant Peptide Mimetic NanoLuc Tracer for Sensitive Immunodetection of Mycophenolic Acid
Source: Anal Chem. 2021 Jul 14;93(29):10358–64. doi: 10.1021/acs.analchem.1c02109 (PMC8478282; doi:10.1021/acs.analchem.1c02109)
Supplement: Supplementary file 1 — ac1c02109_si_001.pdf [file ac1c02109_si_001.pdf]

Recombinant Peptide Mimetic-NanoLuc Tracer for Sensitive Immunodetection of Mycophenolic Acid

Álvaro Luque-Uría[†], Riikka Peltomaa[†], Tarja K. Nevanen,[$]* Henri O. Arola,[$] Kristiina Iljin,[$] Elena Benito-Peña[†]*, María C. Moreno-Bondi[†]*

[†]Chemical Optosensors and Applied Photochemistry Group (GSOLFA), Department of Analytical Chemistry, Faculty of Chemistry, Complutense University of Madrid, 28040 Madrid, Spain. [$]VTT Technical Research Centre of Finland Ltd, Tietotie 2, FI-02150 Espoo, Finland.

Supporting Information

Experimental

Antibody Coupling to Magnetic Beads 2

Antibody Biotinylation 2

Synthetic Peptide-Based ELISA 2

SPR Measurements 3

RRLC-DAD Method 3

Blood Sample Treatment 3

Results

Figure S1 4

Figure S2 4

Table S1 5

Figure S3 5

Figure S4 5

Figure S5 6

Figure S6 6

Figure S7 7

Table S2 7

Table S3 8

Table S4 8

References 9

Experimental

Antibody Coupling to Magnetic Beads

Recombinant anti-MPA Fab, previously described by Tullila and Nevanen,[1] was conjugated to carboxylated paramagnetic beads. Briefly, 4.5 mg of beads were suspended in 150 µL of buffer A (100 mmol L^{-1} sodium phosphate, pH 6.8, 1 mol L^{-1} NaCl, 20% EtOH) and vortexed. The beads were washed three times with water and resuspended in 750 µL of 100 mmol L^{-1} $CoCl_2$. The reaction was incubated for 30 min at room temperature in shaking and subsequently the beads were washed five times with buffer B (20 mmol L^{-1} sodium phosphate, pH 7.0, 0.5 mol L^{-1} NaCl). Then, the beads were mixed with anti-MPA (1 mg of anti-MPA per 2 mg of beads in a total volume of 1 mL) in PBS buffer, and the solution was incubated overnight at +4 °C under rotation. After collecting the beads and aspirating the buffer, 1 mL of 0.03% H_2O_2 in milli-Q water was added to the suspension and incubated for 4 h at room temperature under rotation. Finally, the beads were washed twice with buffer B and stored at +4 °C in the same buffer. Success of the immobilization of the anti-MPA Fab fragments onto the magnetic beads was confirmed by bead ELISA using MPA-alkaline phosphatase enzyme conjugate and para-Nitrophenylphosphate as a substrate for the detection.[21] A total of 260 µg of anti-MPA Fab fragment was bound to the magnetic beads.

Antibody Biotinylation

Anti-MPA Fab antibody (anti-MPA)[1] was biotinylated using EZ-Link Sulfo-NHS-LC-Biotin, according to the manufacturer's instructions,[2] using a 3× molar excess of Sulfo-NHS-LC-Biotin in comparison to the antibody. Purification was carried out with Illustra NAP-5 columns according to the manufacturer's instructions.

Synthetic Peptide-Based ELISA

The amino acid sequence of clone A2 was identified by DNA sequencing and the biotinylated peptide with the sequence A(CEGLYAHWC)GGGSK(Bio)-NH_2 was synthesized at Peptide Synthetics (Fareham, UK). The two C's were bound by a disulfide bridge. GGGS was included in the sequence as a linker, the same as in the phage of origin, and biotin was included in the side chain of C-terminal lysine residue.

In order to optimize the conditions of the assay, a checkerboard-type titration was performed using different concentrations of the biotinylated peptide A2 (A2-bio) and anti-MPA. Briefly, 100 µL of different concentrations of A2-bio (ranging from 0.05 to 5 µg mL^{-1}) dissolved in assay buffer were added to neutravidin-coated clear plates blocked with SuperBlock and incubated during 30 min at room temperature. After washing the wells 3 times with PBS, 100 µL of a solution containing different concentrations of non-biotinylated anti-MPA (ranging from 0.05 to 5 µg mL^{-1}) in the presence and absence of 16 ng mL^{-1} of free MPA (anti-MPA and MPA previously incubated for 15 min at room temperature) in assay buffer were added to the wells and incubated for 30 min at room temperature. Once the plates were washed, HRP-conjugated anti-IgG monoclonal antibody (0.27 µg mL^{-1} in assay buffer; 80 µL per well) was added and incubated for 30 min at room temperature followed by a washing step. Then, 80 µL of TMB was added to the solution, and after 1 min the reaction was stopped with 2 mol L^{-1} H_2SO_4. The absorbance at 450 nm was measured with a CLARIOstar microplate reader

For comparison purposes, a bead-based assay was also developed using a similar strategy as the bead-based assay for the phage-displayed peptide with slight modifications. In this case,

100 µL of a 0.1 µg mL^{-1} bio-A2 solution were added to the neutravidin-functionalized beads solution as described before. After a 30-min incubation and a similar washing step, 100 µL of a solution containing 0.5 µg mL^{-1} of anti-MPA and different concentrations of free MPA, previously incubated during 10 min, were added to the beads and incubated for another 30 min. After washing, an anti-IgG-HRP antibody was added, incubated 30 min and washed thereafter. Finally, 80 µL of Amplex UltraRed was added and the fluorescence was monitored as described above.

SPR Measurements

The binding experiments using Biacore T200 instrument (Cytiva, formely GE Healthcare Life Sciences) and Sensor Chip CM5 with carboxymethylated dextran matrix (Cytiva) were performed at +25 °C. EDC/NHS chemistry was used according to manufacturer's instructions to immobilize anti-mycophenolic acid Fab fragment on active surface (flow channel 2) and anti-ochratoxin A (OTA) Fab fragment on reference surface (flow channel 1). As running buffer, 1× PBS-P (20 mmol L^{-1} phosphate buffer, 2.7 mmol L^{-1} KCl, 0.137 mol L^{-1} NaCl, 0.05% P20, Cytiva) was used. Seven serially diluted concentrations (0, 0.12, 4.9, 19.5, 78.1, 312.5 and 1250 nmol L^{-1}) of cyclic peptide or mycophenolic acid (Sigma-Aldrich, St. Louis, MO, USA) in 1× PBS-P were injected at a flow rate of 20 µL min^{-1} for 2 min. After dissociation phase of 5 min, regeneration was done with 10 mmol L^{-1} NaOH 0.05% P20 (contact time 60 s, flow rate: 30 µL min^{-1}) followed by stabilization period of 60 s. All samples were analyzed in replicates. The results were analyzed with the Evaluation T200 -software with a 1:1 Langmuir binding model.

RRLC-DAD Method

The optimized separation of the three metabolites was performed in gradient elution mode, yielding a resolution (Rs) higher than 2.0 and with retention times (rt) of 5.7, 8.8 and 12.0 min for MPAG, Acyl-MPAG and MPA respectively. The rt were reproducible between runs. **Figure S7** shows a chromatogram of a standard mixture of the metabolites. The three metabolites were quantified using an internal standard (MPA-LuciferYellow) calibration model using a nine-point calibration curve, with a concentration range (0.5–80 ng mL^{-1} for MPA; 50–15000 ng mL^{-1} for MPAG and 12–250 ng mL^{-1} for Acyl-MPAG) for the three metabolites. The r^2 value for standard curves was >0.99 for all compounds tested.

Blood Sample Treatment

To isolate the plasma, blood samples from five different transplanted patients under different treatments (T1–T5) and three healthy patients (H1–H3) were centrifuged at 2000 g for 15 min at room temperature. The supernatant was then transferred to a 1.5 mL tube and 500 µL of that supernatant were added to ultrafiltration 3K Amicon Ultra tubes, previously rinsed with PBST, and centrifuged at 12,045 g for 30 min. The ultrafiltered plasma samples were kept at −20 °C until analyzed. Free MPA was analyzed in plasma samples after a 1:8 dilution in SuperBlock + 0.05% T20.

For the RRLC-DAD analysis, 1000 µL of acetonitrile were added to the ultrafiltered plasma samples and vortexed for 5 minutes at 1000 rpm. The samples were then centrifuged at 12045 g for 10 minutes and the supernatant was transferred to a new tube. The supernatant was evaporated up to circa 30 µL and it was reconstituted in a final volume of 150 µL PBST acidified with 0.4% TFA (final concentration).

Results

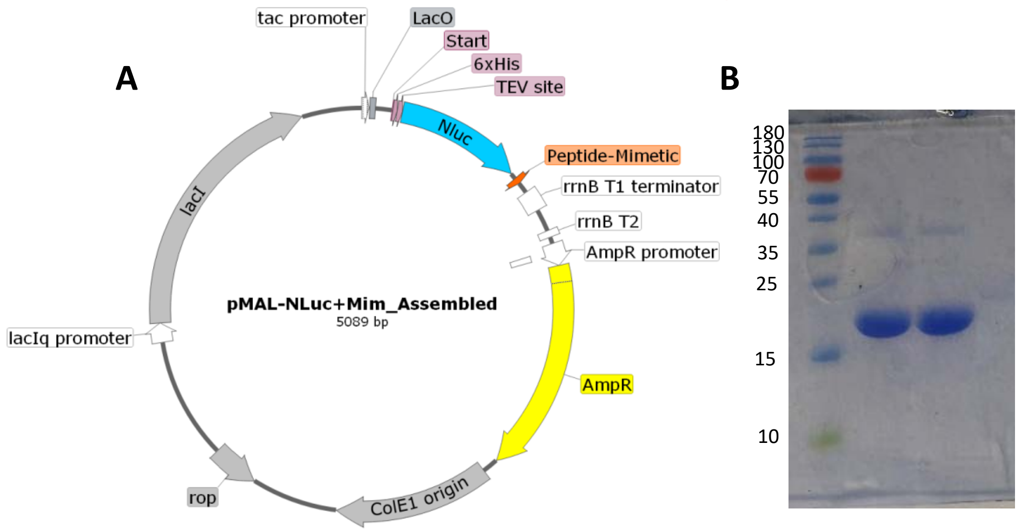

Figure S1. Construction of the NanoLuc-peptide mimetic fusion. (A) Scheme of the fusion protein construct with its main features. (B) SDS-PAGE analysis of the purified fusion protein with Coomassie brilliant blue protein staining: lane 1, molecular marker (Thermo Scientific™ PageRuler™ Prestained Protein Ladder, 10 to 180 kDa); 2, purified product with a 5 minute boiling step at +95 °C; 3, purified fusion protein without a boiling step. The thickest band corresponds to the molecular weight of the fusion protein (22 kDa).

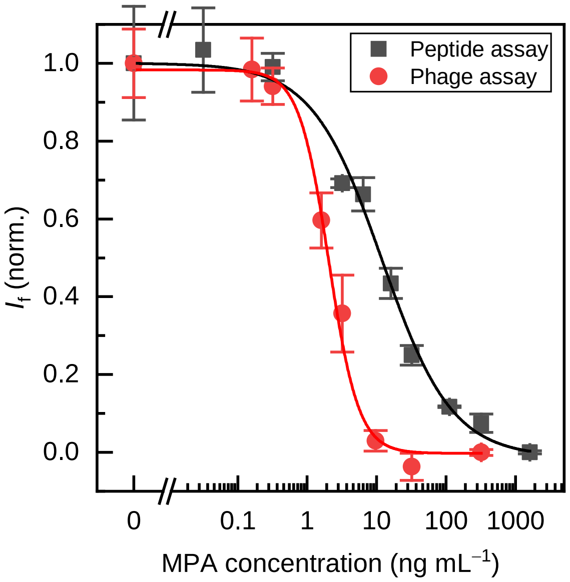

Figure S2. Bead-based immunoassays with the phage clone A2 (red) and the synthetic biotinylated peptide mimetic (black). In both cases, neutravidin-functionalized magnetic beads were used. The fluorescent substrate was also the same in both cases. Fluorescence signals (λ_{ex} = 530 nm, λ_{em} = 590 nm) were measured after adding the Amplex UltraRed substrate. The results are shown as the normalized mean values ± the standard error of the mean (n = 3) with a logistic fit (OriginPro 2019).

Table S1. Kinetic constants determined for anti-MPA (14) Fab interaction with MPA and cyclic peptide (A2) measured by BIAcore. The values present the averages (± SE) obtained with seven analyte (MPA or cyclic peptide (A2)) concentrations.

	**Association rate constant k_a (1/Ms)**	**Dissociation rate constant k_d (1/s)**	**Affinity constant K_D (M)**
MPA	$(1.846 \pm 0.009) \times 10^6$	0.0741 ± 0.0004	4.016×10^{-8}
Cyclic peptide (A2)	$(4.06 \pm 0.02) \times 10^5$	0.583 ± 0.001	1.434×10^{-6}

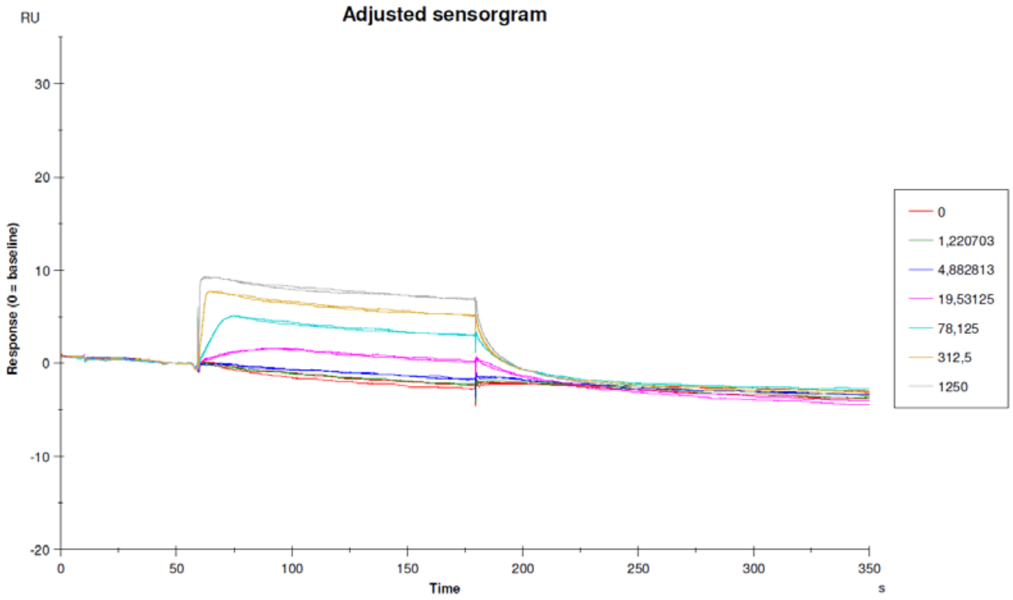

Figure S3. Sensograms presenting mycophenolic acid (MPA) interaction with anti-MPA Fab fragment using Biacore T200. The colors indicate different nanomolar concentrations of MPA.

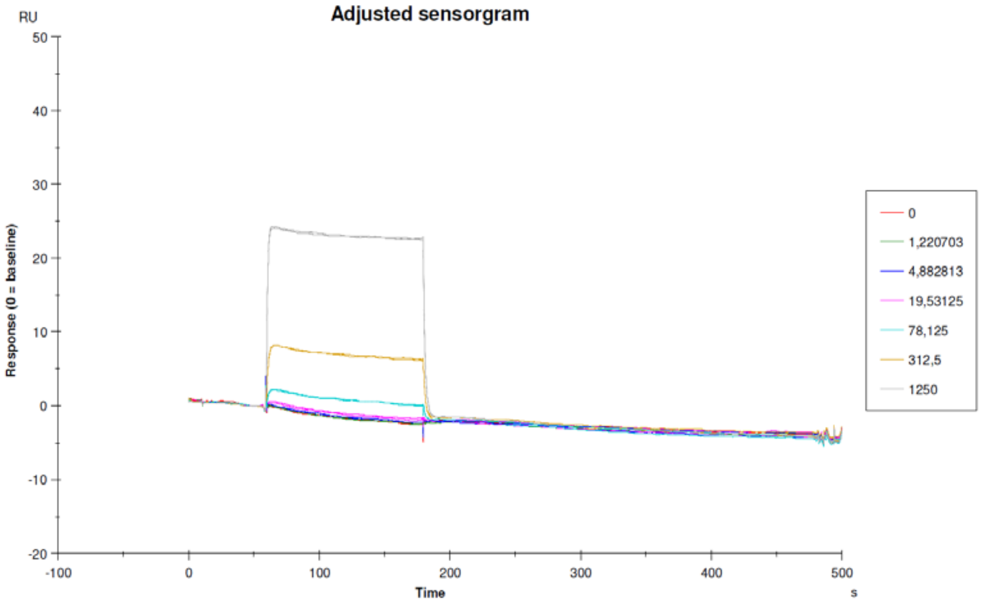

Figure S4. Sensograms presenting cyclic peptide interaction with anti-MPA Fab fragment using Biacore T200. The colors indicate different nanomolar concentrations of cyclic peptide.

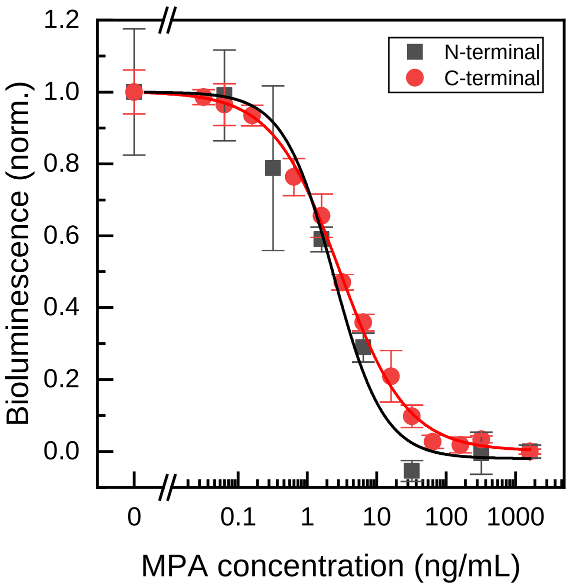

Figure S5. Calibrate comparison between the N-terminal (NanoLuc-A2) and the C-terminal (A2-Nanoluc) fusions, under the same assay conditions described in the main text. The results are shown as the normalized mean values ± the standard error of the mean (*n* = 3) with a logistic fit (OriginPro 2019).

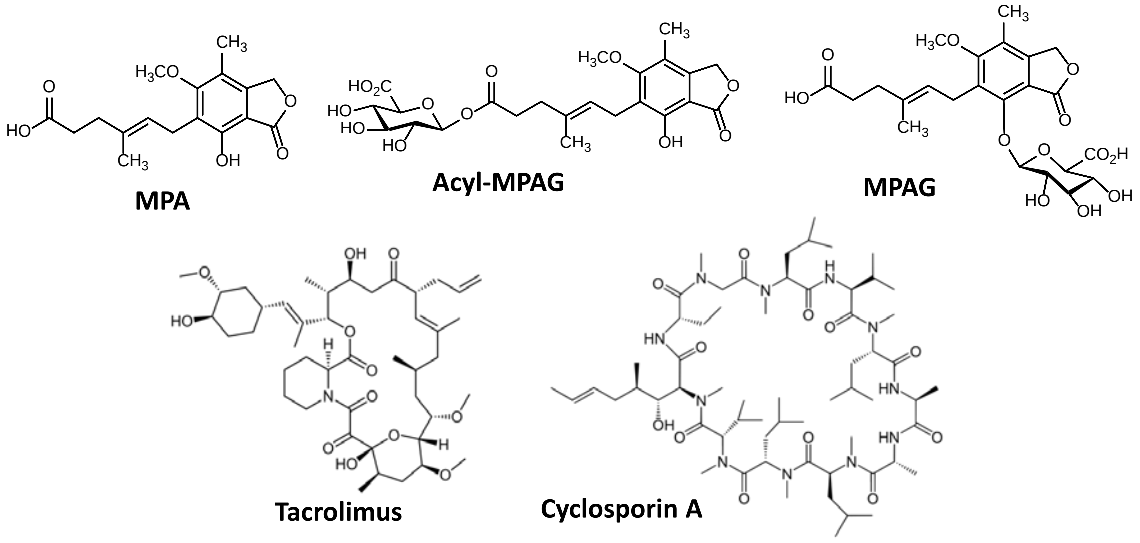

Figure S6. Chemical structures of mycophenolic acid (MPA), its two main metabolites, (Acyl-MPAG and MPAG) and two immunosuppressant drugs commonly co-administered to transplanted patients (Tacrolimus and Cyclosporin A).

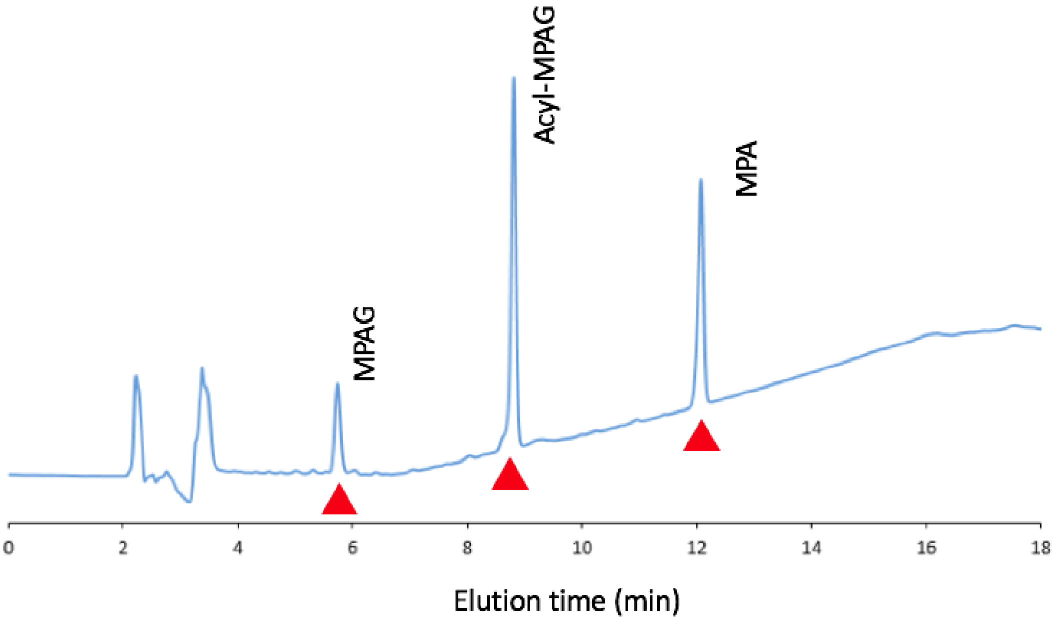

Figure S7. RRLC elution profile of MPA and its metabolites MPAG and acyl-MPAG. Standard solution of MPA prepared at 80 ng mL^{-1}; and MPAG, acyl-MPAG prepared at 250 ng mL^{-1}.

Table S2. Assays comparison for the detection of MPA.

**Method**	**Assay format**	**Detect**	**LOD (ng mL^{-1})**	**Dynamic range (ng mL^{-1})**	**Ref.**
EMIT 2000 MPA	Homogeneous	Total	*n.d.*	100–15000	Siemens
CEDIA MPA	Homogeneous	Total	*n.d.*	300–10000	Thermo Scientific
Roche total MPA (EMIT)	Homogeneous	Total	300	400–15000	Roche
EMIT	Homogeneous	Free	1	10–1250	(3)
PETINIA	Homogeneous	Total	*n.d.*	200–30000	(4)
FPIA	Homogeneous	Free	3.2	6.8–156	(5)
ELISA	Heterogeneous	–	*n.d.*	*n.d.*	(1)
UPLC MS/MS	–	Total	70	200–30000	(6)
HPLC-UV	–	Free	4	60–1000	(7)
HPLC-FLD	–	Total<br>Free	8<br>*n.d.*	50–40000<br>5–1000	(8)
Peptide-Nanoluc	Heterogeneous	Free	0.26	0.64–14	This work
Peptide-Based	Heterogeneous	Free	0.94	2.4–60	This work
Phage-Based	Heterogeneous	Free	0.69	1.0–4.1	This work

n.d. not determined

Table S3. MPAG concentration levels found in the analyzed samples.

Samples	Average, µg/mL	SD, µg/mL	Remarks
T1	1.7	0.0	< LOQ (Biosensor)
T2	12.7	1.3	< LOQ (Biosensor)
T3	4.2	0.2	< LOQ (Biosensor)
T4	4.1	0.6	< LOQ (Biosensor)
T5	0.13	0.2	<LOD (HPLC)
H1	0.02	0.1	<LOD (HPLC)
H2	0.02	0.1	<LOD (HPLC)
H3	-0.04	0.0	<LOD (HPLC)

Table S4. Administered doses of MPA to the analyzed transplanted patients**.**

**Patient**	**Administered drug**	**Dose**
T1	Myfortic	360 mg/12 h
T2	Myfortic	360 mg/12 h
T3	Myfortic	180 mg/8 h
T4	Myfortic	180 mg/8 h
T5	Myfortic	180 mg/8 h

References

(1) Tullila, A.; Nevanen, T. Utilization of Multi-Immunization and Multiple Selection Strategies for Isolation of Hapten-Specific Antibodies from Recombinant Antibody Phage Display Libraries. *IJMS* **2017**, *18*, 1169.

(2) EZ-Link™ Sulfo-NHS-LC-Biotin, formato No-Weigh™ https://www.thermofisher.com/order/catalog/product/A39257 (accessed Jan 25, 2021).

(3) Rebollo, N.; Calvo, M. V.; Martín-Suárez, A.; Domínguez-Gil, A. Modification of the EMIT Immunoassay for the Measurement of Unbound Mycophenolic Acid in Plasma. *Clin Biochem* **2011**, *44*, 260–263.

(4) Dasgupta, A.; Tso, G.; Chow, L. Comparison of Mycophenolic Acid Concentrations Determined by a New PETINIA Assay on the Dimension EXL Analyzer and a HPLC-UV Method. *Clin Biochem* **2013**, *46*, 685–687.

(5) Glahn-Martínez, B.; Benito-Peña, E.; Salis, F.; Descalzo, A. B.; Orellana, G.; Moreno-Bondi, M. C. Sensitive Rapid Fluorescence Polarization Immunoassay for Free Mycophenolic Acid Determination in Human Serum and Plasma. *Anal Chem* **2018**, *90*, 5459–5465.

(6) Reséndiz-Galván, J. E.; Romano-Aguilar, M.; Medellín-Garibay, S. E.; Milán-Segovia, R. del C.; Chevaile-Ramos, A.; Romano-Moreno, S. Determination of Mycophenolic Acid in Human Plasma by Ultra-performance Liquid Chromatography–Tandem Mass Spectrometry and Its Pharmacokinetic Application in Kidney Transplant Patients. *Biome Chromatogr* **2019**, *33*.

(7) Aresta, A.; Palmisano, F.; Zambonin, C. G.; Schena, P.; Grandaliano, G. Simultaneous Determination of Free Mycophenolic Acid and Its Glucuronide in Serum of Patients under Mycophenolate Mophetil Therapy by Ion-Pair Reversed-Phase Liquid Chromatography with Diode Array UV Detection. *J Chromatogr B* **2004**, *810*, 197–202.

(8) Shen, J.; Jiao, Z.; Yu, Y.-Q.; Zhang, M.; Zhong, M.-K. Quantification of Total and Free Mycophenolic Acid in Human Plasma by Liquid Chromatography with Fluorescence Detection. *J Chromatogr B* **2005**, *817*, 207–213.
